# Dynamics of the Two Heterochromatin Types during Imprinted X Chromosome Inactivation in Vole *Microtus levis*

**DOI:** 10.1371/journal.pone.0088256

**Published:** 2014-02-04

**Authors:** Evgeniya A. Vaskova, Elena V. Dementyeva, Alexander I. Shevchenko, Sophia V. Pavlova, Elena V. Grigor'eva, Antonina I. Zhelezova, John L. VandeBerg, Suren M. Zakian

**Affiliations:** 1 Institute of Cytology and Genetics, Siberian Branch of the Russian Academy of Sciences, Novosibirsk, Russia; 2 State Research Institute of Circulation Pathology, Novosibirsk, Russia; 3 Institute of Chemical Biology and Fundamental Medicine, Siberian Branch of the Russian Academy of Sciences, Novosibirsk, Russia; 4 Department of Genetics and Southwest National Primate Research Center, Texas Biomedical Research Institute, San Antonio, Texas, United States of America; CNRS, France

## Abstract

In rodent female mammals, there are two forms of X-inactivation – imprinted and random which take place in extraembryonic and embryonic tissues, respectively. The inactive X-chromosome during random X-inactivation was shown to contain two types of facultative heterochromatin that alternate and do not overlap. However, chromatin structure of the inactive X-chromosome during imprinted X-inactivation, especially at early stages, is still not well understood. In this work, we studied chromatin modifications associated with the inactive X-chromosome at different stages of imprinted X-inactivation in a rodent, *Microtus levis*. It has been found that imprinted X-inactivation in vole occurs in a species-specific manner in two steps. The inactive X-chromosome at early stages of imprinted X-inactivation is characterized by accumulation of H3K9me3, HP1, H4K20me3, and uH2A, resembling to some extent the pattern of repressive chromatin modifications of meiotic sex chromatin. Later, the inactive X-chromosome recruits trimethylated H3K27 and acquires the two types of heterochromatin associated with random X-inactivation.

## Introduction

In mammals, there are three forms of X-chromosome inactivation (XCI) - meiotic, imprinted and random. The first form, meiotic sex chromosome inactivation (MSCI), occurs during spermatogenesis in eutherians and marsupials at the pachytene stage when all homologous chromosomes are paired with synaptonemal complexes. Heteromorphic X- and Y-chromosomes become transcriptionally inactive and form so-called sex or XY-body [1], [2], [3], [4]. The transcriptional suppression initiated by MSCI does not end with meiosis I, but is sustained in meiosis II and is manifested as postmeiotic sex chromatin (PMSC) during spermiogenesis [5]. The other two forms take place in females. During imprinted XCI the paternally inherited X-chromosome is inactivated in all of the tissues of marsupials [6] and extraembryonic tissues (placenta, yolk sac) of some eutherians (e.g., rodents) [7], [8], [9]. In eutherian embryonic tissues, XCI is random. However, random XCI occurs in both embryonic and extraembryonic tissues in human [10].

The inactive X-chromosome (Xi) in somatic cells and extraembryonic endoderm (XEN) cells which represent random and imprinted XCI, respectively, is depleted in the modifications characteristic of active chromatin. The modifications associated with the inactive chromatin are distributed along Xi as discrete bands and form two types of facultative heterochromatin [11], [12], [13], [14], [15], [16], [17], [18]. The first type corresponds to gene-rich G-light bands. It consists of nuclear *Xist* RNA playing a key role in XCI and *Xist*-dependent chromatin modifications such as trimethylated H3K27 (H3K27me3) and monoubiquitylated H2AK119 (uH2A). The first type of facultative heterochromatin is detected predominately on Xi and is involved in gene silencing. Its formation is closely linked with *Xist* RNA spreading. The second type of facultative heterochromatin is revealed in the repeat rich Xi regions corresponding to G-dark bands. It contains the other set of chromatin modifications: heterochromatic protein HP1, trimethylated H3K9 (H3K9me3) and H4K20 (H4K20me3). This type of facultative heterochromatin is observed not only on Xi but also in the constitutive heterochromatin of other chromosomes. However, the dynamics of the two types of facultative heterochromatin at early stages of imprinted XCI as well as the mechanisms underlying imprinted XCI are still poorly understood.

In the study, we examined the dynamics of chromatin modifications associated with both types of Xi facultative heterochromatin in female trophoblast stem (TS) cells as well as preimplantation and postimplantation embryos of the common vole, *Microtus levis*. TS cells are the first specialized cell lineage of preimplantation embryos and demonstrate imprinted XCI. We found out that imprinted XCI occurred in two steps. Xi at early stages of imprinted XCI was enriched with repressive modifications H3K9me3, HP1, H4K20me3, and uH2A. Further, this pattern changed by recruiting H3K27me3 and became similar to that of Xi in somatic and XEN cells.

## Results

### Xi chromatin modifications in trophoblast stem cells of the vole, *M. levis*

**Figure 1:**
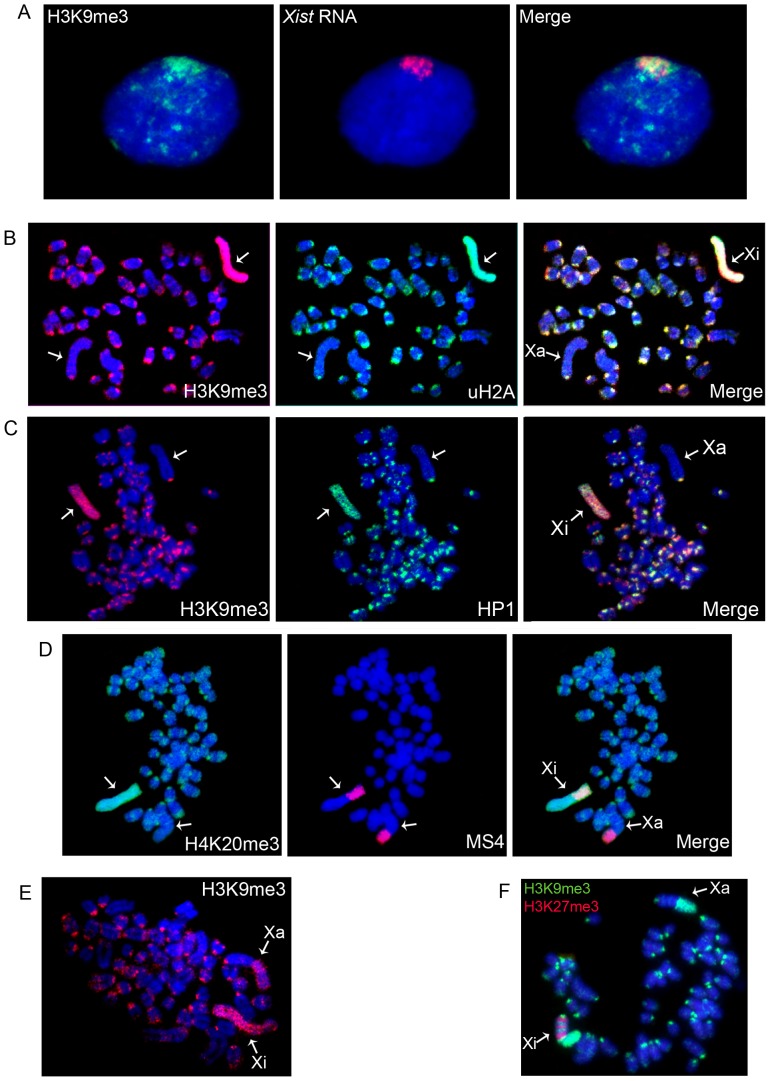
Distribution of repressive chromatin modifications on metaphase chromosomes from vole undifferentiated TS cells. (**A**) Immunostaining (H3K9me3, green) combined with RNA FISH (*Xist* RNA, red); (**B**) H3K9me3 (red) and uH2A (green); (**C**) H3K9me3 (red) and HP1 (green); (**D**) Immunostaining (H4K20me3, green) combined with DNA FISH (MS4 repeat, red); (**E**) An example of repressive chromatin modification localization in the heterochromatic block of the active X-chromosome, H3K9me3 (red); (**D**) An example of repressive chromatin modification distribution in XEN cells. H3K27me3 (red) and H3K9me3 (green). Metaphase spreads were counterstained with DAPI (blue). Active (Xa) and inactive (Xi) X-chromosomes are indicated by arrows.

First we examined Xi chromatin modifications in vole TS cells described in our previous publication [19]. The XX TS cell line (R1) was derived and maintained in the absence of FGF-4 and heparin, a condition that maintains the undifferentiated state of multipotent TS cells in mouse [20]. In vole extraembryonic tissues, the paternal X-chromosome is inactivated [21], [22] and the vole TS cells, like these of mice, demonstrate imprinted XCI and *Xist* RNA accumulation on Xi (Figure 1A) [19]. The X-chromosome of *M. levis* is acrocentric and has a giant block of constitutive heterochromatin consisting of two repetitive sequences: MS3 (3265 bp) and MS4 (4088 bp), which contain mobile element fragments [23].

In mouse XX TS cells, Xi is known to be enriched with H3K27me3 and transiently associated with Polycomb repressive complex 2 (PRC2) which mediates H3K27 trimethylation [24]. We expected to observe the same Xi chromatin structure in vole XX TS cells. Surprisingly, immunostaining of metaphase chromosomes with antibodies to H3K27me3 did not reveal this modification on Xi in vole undifferentiated TS cells. We also were not able to detect the EED protein, which is a subunit of PRC2 complex found on Xi in mouse (data not shown). Instead, we observed that Xi in vole undifferentiated TS cells was enriched with H3K9me3, HP1, H4K20me3, and uH2A, which were uniformly distributed along the entire Xi in contrast to somatic and XEN cells (Figure 1). These modifications were also detected in the constitutive heterochromatin of pericentromeric and telomeric regions. Interestingly, H3K9me3, H4K20me3, and HP1 were revealed as well in the heterochromatic block of the active X-chromosome but only in approximately 50 of 100 metaphase spreads analyzed (Figure 1E).

To eliminate a possibility that the differences in Xi chromatin structure between mouse and vole XX TS cells are due to different derivation and maintenance conditions, we obtained a new vole TS cell line according to the standard mouse TS cell protocol using FGF-4 and heparin. We found that these vole XX TS cell lines had the same gene expression and differentiation pattern as the R1 cell line (Figure S1). As in the R1 line, Xi in these undifferentiated TS cells was enriched with H3K9me3, HP1, H4K20me3, and uH2A, while both EED and H3K27me3 were not detected (Figure S2).

**Figure 2:**
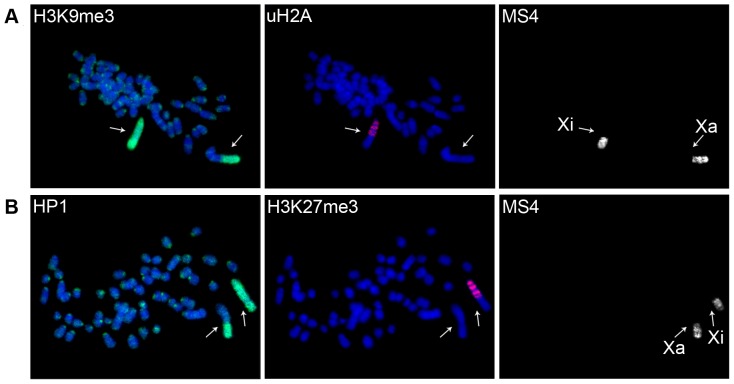
Accumulation and redistribution of repressive chromatin modifications during vole TS cell differentiation. (**A**) uH2A forms distinct bands on Xi and is excluded from heterochromatic regions of Xi and other chromosomes. Immunostaining (uH2A, red and H3K9me3, green) combined with DNA FISH (MS4 repeat); (**B**) H3K27me3 accumulation on Xi. Immunostaining with antibodies to H3K27me3 (red) and HP1 (green) combined with DNA FISH (MS4 repeat). Metaphase spreads were counterstained with DAPI (blue). Active (Xa) and inactive (Xi) X-chromosomes are indicated by arrows.

**Figure 3:**
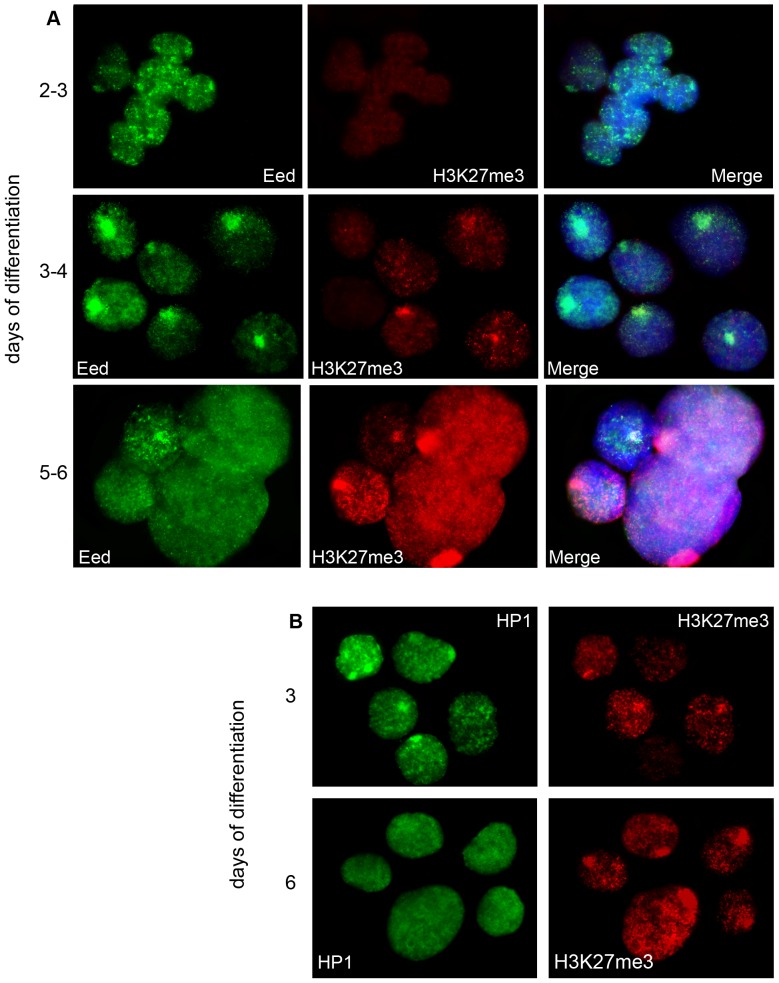
Dynamics of repressive chromatin modification during vole TS cell differentiation. (**A**) Localization of Eed and H3K27me3 on the inactive X-chromosome at different stages of TS cell differentiation; (**B**) H3K27me3 accumulation on Xi is accompanied by a significant decrease in HP1. Immunostaining with antibodies to HP1 (green) and H3K27me3 (red) at different stages of TS cell differentiation.

In addition, we found that the pattern of the repressive modifications on Xi changed during differentiation of both types of vole TS cell lines. uH2A formed distinct bands on Xi and was excluded from heterochromatic regions of Xi and other chromosomes (Figure 2A). On the fourth day of differentiation we observed H3K27me3 enrichment on Xi (Figure 2B, 3A). By the fifth or sixth day, enrichment was detected in almost all TS cells. H3K27me3 accumulation on Xi was accompanied by a significant decrease in H3K9me3, HP1, and EED recruitment (Figure 3, Table S1).

Thus, we have shown that H3K9me3, HP1, H4K20me3, and uH2A form the structure of Xi at early stages of imprinted XCI in vole *M. levis*. These modifications are uniformly distributed along Xi, and this pattern does not depend on the way of derivation and cultivation of vole TS cells. A similar distribution pattern of H3K9me3, HP1, and uH2A is characteristic of the XY-body in MSCI, which prompted us to investigate MSCI and PMSC in *M. levis*.

### The XY-body chromatin modifications at the meiotic and post-meiotic stages in vole spermatogenesis

**Figure 4:**
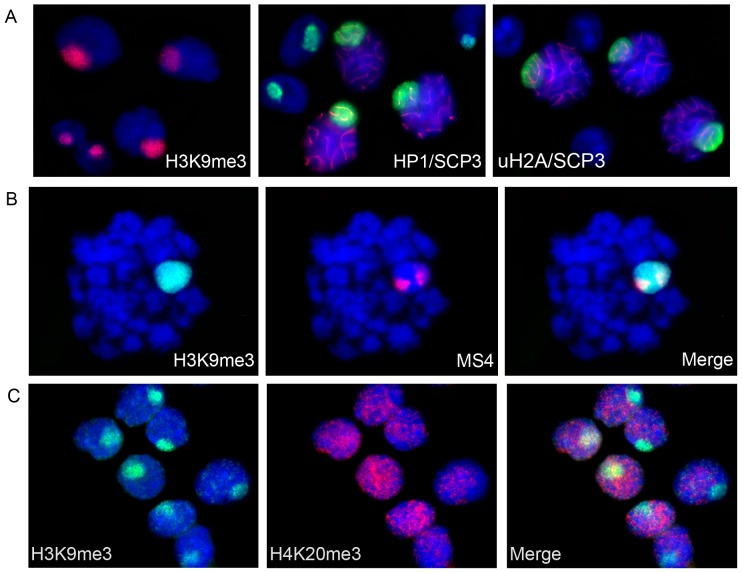
The XY-body chromatin modifications at the meiotic and postmeiotic stages in vole spermatogenesis. (**A**) Co-immunostaining of H3K9me3 (red), HP1 (green), uH2A (green) with antibodies to SCP3 (red) (marker of pachytene stage) was used to distinguish between spermatocytes I and round spermatids; (**B**) Immunostaining (H3K9me3, green) combined with DNA FISH (MS4 repeat, red); (**C**) Co-immunostaining of H3K9me3 (green) and H4K20me3 (red). Nuclei were counterstained with DAPI (blue).

The study of histone modification dynamics during MSCI has shown conservation of the process in eutherians and marsupials [2], [5], [25]. We examined the chromatin of X- and Y-chromosomes during vole spermatogenesis. Co-immunostaining with antibodies to SCP3 (synaptonemal complex protein 3) and inactive chromatin modifications (H3K9me3, H3K27me3, uH2A, H4K20me3, and HP1) was used. SCP3 staining allowed us to mark the pachytene stage when MSCI is known to occur in eutherians and marsupials. Despite the fact that sex chromosomes of *M. levis* never pair and thus do not form a true synaptonemal complex [26], we detected a condensed chromatin structure at the nuclear periphery, the so-called sex or XY-body. The XY-body was enriched with uH2A, H3K9me3, and HP1, implying that MSCI did take place at the pachytene stage in *M. levis* (Figure 4). At postmeiotic stages, X- and Y-chromosomes were still DAPI-intense and associated with the inactive chromatin modifications. PMSC at the round spermatid stage in *M. levis* lost uH2A but retained H3K9me3 and HP1. It is worth noting that H3K27me3 and H4K20me3 demonstrated no enrichment on the XY-body, and these modifications were not present at all in some nuclei (Figure 4C).

Our results have demonstrated that MSCI occurs during vole spermatogenesis and that transcriptional silencing spreads to PMSC. They also demonstrated that the chromatin structure of sex chromosomes is formed by H3K9me3, HP1, and, at some stages, by uH2A.

### The Y-chromosome chromatin in vole trophoblast stem cells

MSCI and postmeiotic silencing affect both X- and Y-chromosomes. We studied distribution of uH2A on metaphase spreads of vole XY TS cells to check whether the Y-chromosome had the same chromatin structure as Xi. uH2A was used in the experiment because H3K9me3, HP1, and H4K20me3 are attributable to constitutive heterochromatin as well and are not restricted to the Y-chromosome in different cell types.

**Figure 5:**
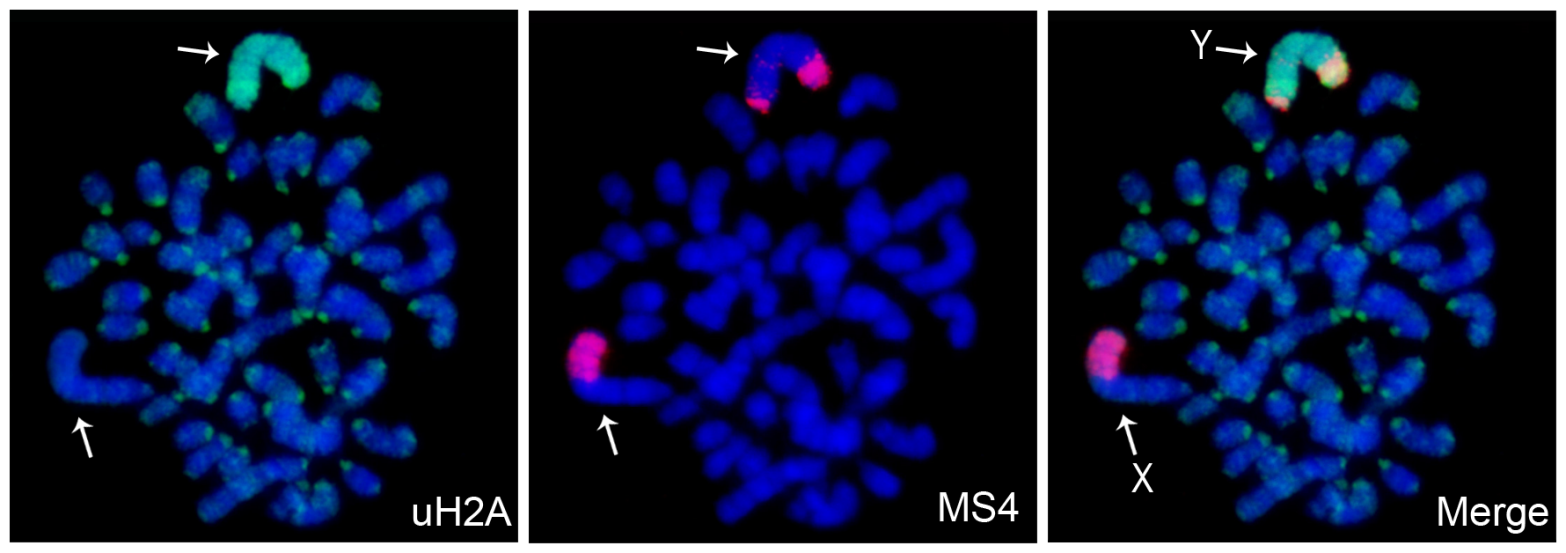
The Y-chromosome chromatin in vole trophoblast stem cells. Immunofluorescence (uH2A, green) combined with DNA FISH (MS4 repeat, red). Metaphase spreads were counterstained with DAPI (blue). X-chromosome (X) and Y-chromosome (Y) are indicated by arrows.

The Y-chromosome of *M. levis* is an acrocentric completely heterochromatinized chromosome. It is easily revealed by strong telomeric and narrow pericentromeric bands of MS4 repeat [23]. uH2A immunostaining combined with DNA FISH (MS4 repeat) showed that the Y-chromosome was enriched with uH2A distributed uniformly along the entire chromosome (Figure 5). In addition, uH2A was observed in the regions of constitutive heterochromatin on autosomes. Therefore, Y-chromosome in vole XY TS cells exhibits the same pattern of uH2A distribution as Xi in XX TS cells.

### Xi chromatin modifications during vole embryogenesis

**Figure 6:**
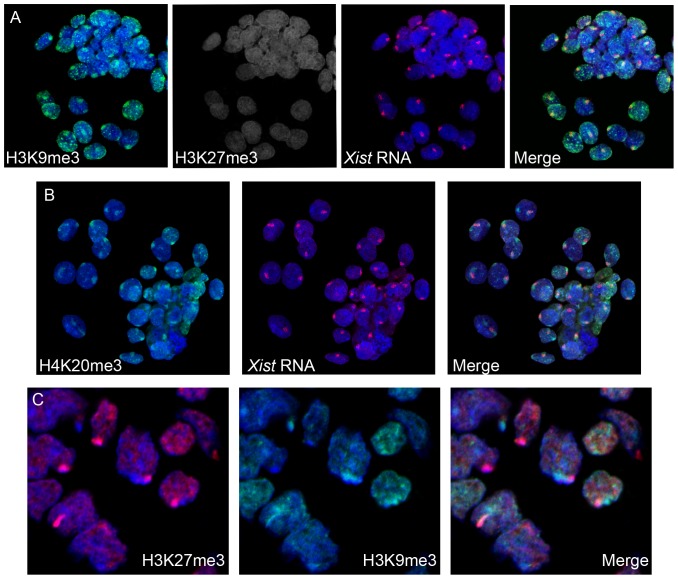
Repressive chromatin modifications at early stages of imprinted XCI during vole preimplantation and postimplantation development. (**A**) Immunofluorescence (H3K9me3, green and H3K27me3, grey) combined with RNA FISH (*Xist* RNA, red) at the blastocyst stage. (**B**) Immunofluorescence (H4K20me3, green) combined with RNA FISH (*Xist* RNA, red) at the blastocyst stage. (**C**) Immunostaining (H3K27me3, red and HP1, green) of extraembryonic ectoderm of 7,5-day cryosectioned embryos.

To verify our findings on Xi chromatin structure in vole XX TS cells, we studied H3K9me3, HP1, H4K20me3, uH2A and H3K27me3 patterns in vole preimplantation development (the blastocyst stage). Female embryos were determined by the presence of *Xist* transcript. In most blastomeres, a strong focus of H3K9me3, H4K20me3, and HP1 co-localizing with the *Xist* RNA signal was detected (Figure 6A, B). In some blastomeres, there was an additional smaller signal. The strong signal corresponded to Xi whereas the small signal might correspond to the block of constitutive heterochromatin on Xa. Interestingly, in some blastomeres from the inner cell mass, there was no enrichment of H3K9me3, H4K20me3, and HP1 on X chromosomes. This may be due to the fact that imprinted XCI is reversed in the inner cell mass, specifically in the cells that give rise to embryo proper [27], [28].

H3K27me3 enrichment typical of Xi in mouse blastocyst was not revealed at this stage in vole (Figure 6A). Surprisingly, we were able to detect H4K20me3, which demonstrated the same distribution pattern as H3K9me3 and HP1. However, the modification was shown not to be associated with Xi in mouse blastocyst [29]. To reveal uH2A, we used FK2 antibodies to polyubiquitylated proteins that produced strong signals over the whole blastomeres so uH2A signals specific for Xi could not be observed. In case of TS cell metaphase spreads and nuclei, an accurate uH2A detection on Xi was possible due to using hypotonic treatment and cytospin technique. Thus, Xi chromatin in vole preimplantation development is enriched with H3K9me3, HP1, and H4K20me3 but not with H3K27me3 and is very similar to that in undifferentiated vole XX TS cells.

Next, the chromatin modification pattern in vole postimplantation development was established. In mouse, implantation is accompanied by reactivation of the paternal X-chromosome in inner cell mass and maintenance of imprinted XCI in trophectoderm and its derivatives [28]. Later stages of imprinted XCI in vole were analyzed in extraembryonic ectoderm of 7,5-day cryosectioned embryos. H3K27me3 accumulation was revealed in all extraembryonic ectoderm cells of some embryos and allowed males to be distinguished from females. In female extraembryonic ectoderm, there were no foci of H3K9me3, HP1, and H4K20me3. Therefore, the vole Xi at postimplantation stages was characterized by H3K27me3 recruitment and reduced levels of H3K9me3, HP1, and H4K20me3 (Figure 6C). This pattern was different from that during preimplantation development but reminiscent of that in differentiated vole XX TS cells.

In summary, Xi chromatin structure at the early stages of imprinted XCI in vole is provided by H3K9me3, H4K20me3, and HP1, which are involved in constitutive heterochromatin formation. Some of the chromatin modifications are also found in the XY-body during MSCI. Later, H3K27me3 is recruited to Xi, and the level of these modifications is reduced. The data on Xi chromatin structure during vole embryogenesis are in agreement with our findings for TS cells, confirming that vole XX TS cells are a useful model system to study imprinted XCI.

## Discussion

In this work, we studied modifications associated with the inactive chromatin at different stages of imprinted XCI in TS cells, as well as during spermatogenesis and early embryonic development of the common vole, *M. levis*.

Xi in vole undifferentiated TS cells derived under various conditions was found to contain repressive chromatin markers H3K9me3, HP1, H4K20me3, and uH2A distributed uniformly, while H3K27me3, a well known component of Xi chromatin in mouse TS cells, was not detected. It has been previously shown that two distinct types of facultative heterochromatin are present on the vole Xi at late stages of both imprinted XCI in XEN cells and random XCI in adult cells [18]. One Xi heterochromatin type is enriched with H3K27me3 and uH2A and located in gene-rich regions, whereas the other one consists of H3K9me3, HP1, and H4K20me3 and is present in gene-poor repeat containing regions. The two heterochromatin types are also found on Xi in adult cells of humans and cattle [11], [17]. Thus, Xi chromatin organization in undifferentiated TS cells differs from that in cells demonstrating later stages of imprinted XCI and random XCI. The distribution pattern of the Xi repressive modifications (except for H4K20me3) in undifferentiated vole TS cells was similar to mammalian XY-body chromatin during MSCI [25], [30], [31], [32]. This is confirmed by our study of the chromatin modifications during spermatogenesis in *M. levis*. Three of the modifications, H3K9me3, HP1, and H4K20me3, are known to be permanent components of constitutive heterochromatin and observed not only on Xi but also in heterochromatic centromeric and telomeric regions of the other chromosomes in vole TS cells. Surprisingly, uH2A was also revealed together with H3K9me3, HP1, and H4K20me3 in the repeat containing heterochromatic regions of chromosomes, suggesting that histone ubiquitylation is not specific for X-chromosome silencing only but can also take part in constitutive heterochromatin formation at certain developmental stages. Thus, it appears that heterochromatin repression with H3K9me3, HP1, H4K20me3, and uH2A at early developmental stages in vole is a genome-wide mechanism, rather than being specific to the paternal X-chromosome.

During TS cell differentiation uH2A becomes restricted only to gene-rich G-light bands of Xi, in which H3K27me3 appears while the level of H3K9me3, HP1, and H4K20me3 on Xi reduces. Therefore, Xi chromatin during TS cell differentiation can be reorganized into the two alternative heterochromatin types described previously, as it is at later stages of imprinted XCI in vole XEN cells.

TS cells are a cell lineage derived from preimplantation embryos and considered to be a model system to examine the process of imprinted XCI. To confirm the data obtained using vole TS cells, chromatin modifications during vole embryonic development were studied. In vole blastocysts corresponding to early stages of imprinted XCI, Xi was associated with H3K9me3, HP1, and H4K20me3, as are vole undifferentiated XX TS cells. In extraembryonic ectoderm of 7,5-day embryos reflecting later stages of imprinted XCI, the chromatin modification pattern on Xi was characterized by H3K27me3 enrichment and reduced levels of H3K9me3, HP1, and H4K20me3, and was similar to that in differentiated XX TS cells. Thus, TS cells reflect properly the events taking place during imprinted XCI in early embryogenesis and can be used to investigate the process.

XCI in mammalian preimplantation development was shown to occur in a species-specific manner [10]. In mouse, H4K20me3 was not found at all in heterochromatic regions at preimplantation stages. However, we detected this modification in constitutive heterochromatin and even on Xi in both vole TS cells and blastocysts. This may imply an earlier involvement of H4K20me3 in imprinted XCI in vole. Conversely, H3K27me3 accumulation could be revealed on the vole Xi only at later stages of imprinted XCI – in extraembryonic ectoderm and differentiated TS cells - suggesting slower dynamics of the modification by comparison with mouse.

Imprinted XCI in mouse was demonstrated to be established in two steps. The first step is a silencing of repeats which occurs independently of *Xist* at the 2-cell stage, while the genes of the paternal X-chromosome remain active [33], [34], [35], [36], [37]. The second step is *Xist*-dependent gene silencing which occurs gradually in preimplantation development. The inactive state of repeat elements is proposed to be inherited from the paternal germline, whereas the genic silencing is established *de novo* in imprinted XCI [34], [36]. Two different states of Xi heterochromatin found during imprinted XCI in vole might correspond to the two steps of imprinted XCI revealed in mouse. If so, the pattern of Xi modifications at early stages of imprinted XCI may represent a mechanism required for silencing that involves repeat elements, and Xi chromatin at later stages of imprinted XCI may correspond to the gene silencing. Interestingly, the repressive chromatin modifications are uniformly distributed along the vole Xi including both gene- and repeat-rich regions, although only repeats are silenced at first steps of imprinted XCI. Taking into account that the maternal X-chromosome is two-fold up-regulated beginning at the zygote stage [38], it is tempting to speculate that the set of modifications also take part in decreasing gene expression on the paternal X-chromosome to achieve a balance between expression levels of X-linked and autosomal genes, which is important during mammalian ontogenesis [39].

It is believed that there is an imprint protecting the maternal X-chromosome from inactivation in mouse preimplantation development [40]. In our study, we found that the active maternal X-chromosome was often depleted in repressive chromatin modifications even in the large block of constitutive heterochromatin in vole TS cells and blastocysts of both sexes. We suggest that the imprint on the maternal X-chromosome may be common to both genes and repeats and protect them from silencing. Therefore, formation of a heterochromatic block on the active X-chromosome is a late event by comparison with the formation of other heterochromatic regions of the genome.

The two types of heterochromatin that determine early and later stages of imprinted XCI in vole are differently presented on Xi during the XCI process in mammals. In marsupials, mammals that have paternally imprinted XCI in all tissues [6], Xi is associated with the modifications of the second heterochromatin type (H3K9me3, H4K20me3, and HP1) whereas accumulation of the modification belonging to the first heterochromatin type (H3K27me3) is only transient and labile [41], [42], [43]. At the same time, only the first heterochromatic type was found on the mouse Xi while the second type is believed not to exist [41]. Both heterochromatic types were identified as non-overlapping bands on Xi during random XCI in human, cattle, and vole [11], [17], [18]. The two heterochromatic types seem to be linked to the two systems of silencing on mammalian Xi but their role in gene repression during imprinted and random XCI needs to be further investigated.

## Materials and Methods

### Ethics statement

The study was carried out according to “The Guidelines for Manipulations with Experimental Animals.” The study was approved by the Ethical Committee of the Institute of Cytology and Genetics, Novosibirsk, permit number: (order of the Presidium of the Russian Academy of Sciences of April 02, 1980 no. 12000-496).

### Cell cultures

Two XX TS cell lines (R1, V1) and one XY TS cell line (R3) of *M. levis* were used. R1 and R3 TS cell lines were described previously [19]. V1 TS cell line was derived and maintained according to the standard protocol for mouse [43].

All of the TS cell lines were grown on a feeder layer of mitotically inactivated embryonic mouse fibroblasts at 37°C in an atmosphere of 5% CO_2_. The V1 cell line was cultivated in RPMI 1640 medium supplemented with 20% fetal bovine serum, 25 ng/ml human recombinant FGF4 (Sigma), 1 µg/ml heparin (Sigma), 2 mM L-glutamine, 1 mM sodium pyruvate, 0.1 mM β-mercaptoethanol, 50 mg/ml penicillin/streptomycin. The R1 and R3 cell lines were maintained in 1∶1 mixture of Dulbecco's modified Eagle's medium (DMEM) and Ham's F12 medium supplemented with 15% fetal bovine serum, 2 mM L-glutamine, 1 mM nonessential amino acids, 0.1 mM β-mercaptoethanol, 50 mg/ml penicillin/streptomycin. All reagents were from Life Technologies if another supplier is not stated.

R1 TS cell line was differentiated by cell cultivating without feeder layer on wells coated with 0.1% gelatin. V1 line was differentiated by removal of FGF4 and heparin from the growth medium.

### Slide preparation and immunostaining

Seminiferous tubulus from adult *M. levis* (30–40 days) were dissected in PBS on ice into segments representing different stages of the spermatogenic cycle. Cells were hypotonized in 0.2% KCl and 0.2% sodium citrate for 5 min at room temperature. Samples (0.1 ml) of the hypotonic cells suspension were cytospun (Citospin2, Shandon) onto clean glass slides at 1000 rpm for 10 min. The slides were treated with Triton X-100 for 5 min at 4°C, rinsed in PBS, and fixed in 4% PFA for 10 min at room temperature.

Analysis of preimplantation embryos was performed as described [44]. The samples were treated with 1% PFA/0,5% Triton X-100 on ice for 5 min, then transferred to 1% PFA on ice for 5 min.

7,5-day vole embryos were fixed overnight at 4°C in 4% PFA, washed 3 times in PBS, and soaked overnight at 4°C in PBS containing 30% (w/v) sucrose. The samples were frozen at −20°C in TissueTek OCT Compound (Sakura), sectioned at 10 µm using a cryostat MICROM HM 505 N, and deposited on polysine slides. Metaphase spread preparation and immunostaining were performed as described previously [18]. Primary and secondary antibodies are listed in Table 1.

**Table 1:** Primary and secondary antibodies used in immunofluorescence assay.

Antibodies	Raised/Type	Source	Cat. No.	Dilution
Primary antibodies				
Anti SCP3	Rabbit polyclonal	Abcam	ab15093	1∶300
Anti H3K9me3	Mouse monoclonal	Abcam	ab6001	1∶200
Anti H3K9me3	Rabbit polyclonal	Upstate (Millipore)	07-442	1∶200
Anti H3K27me3	Rabbit polyclonal	Upstate (Millipore)	05-851	1∶500
Anti HP1 γ	Mouse monoclonal	Upstate (Millipore)	05-689	1∶300
AntiCBX1/HP1β	Rat monoclonal	Abcam	ab10811	1∶5
Anti ubiquitin-protein conjugates (FK2)	Mouse monoclonal	BIOMOL International, LP	PW 8810	1∶300
Anti H4K20me3	Rabbit polyclonal	Abcam	ab9053	1∶500
Secondary antibodies				
Alexa Fluor 568 goat anti mouse IgG (H+L) highly cross adsorbed	Made in goat	Molecular Probe (Invitrogen)	A11031	1∶400
Alexa Fluor 488 goat anti mouse IgG (H+L) highly cross adsorbed	Made in goat	Molecular Probe (Invitrogen)	A11029	1∶400
Alexa Fluor 568 goat anti rabbit IgG (H+L)	Made in goat	Molecular Probe (Invitrogen)	A11011	1∶400
Alexa Fluor 488 goat anti rabbit IgG (H+L)	Made in goat	Molecular Probe (Invitrogen)	A11008	1∶400
Alexa Fluor 350 goat anti mouse IgG (H+L	Made in goat	Molecular Probe (Invitrogen)	A11045	1∶400

The nuclei and chromosomes were visualized using a Nikon X 100 fluorescence microscope and Imstar software. No less than 100 metaphase spreads were analyzed for each chromatin modification. The preimplantation and postimplantation vole embryos were visualized using a laser scanning microscope LSM 780 (Zeiss) in the centre of microscopic analysis at the Institute of Cytology and Genetics.

### Sequential IF/DNA FISH, RNA FISH

Immunofluorescence was carried out first. A plasmid containing the MS4 repeat was used as a probe for DNA FISH and labeled by nick translation with biotin-16-dUTP (Roche). Phage S6 containing promoter region and exons 1–4 of vole *Xist* [45] was used as a probe for RNA FISH and labeled directly with Cy3. Prior to DNA FISH, slides were fixed in 4% paraformaldehyde in PBS for 10 min at room temperature. Then slides were treated with 0.1M HCl/0.7% Triton for 10 min on ice and denatured in 70% formamide/2xSSC for 20 minutes at 70°C. After an overnight hybridization at 37°C, the preparations were washed with 50% formamide/2xSSC at 45°C, 2xSSC at 40°C. Biotinylated probes were detected using streptavidin-Cy3 or fluorescein-avidin/anti-avidin system (Vector Laboratories). RNA FISH was performed as described [46], [47]. Preparation analysis was carried out as for IF.

### Vole TS cell characterization

RT-PCR analysis, isolation and analysis of tumors of V1 TS cells were performed as described previously [19], [21].

## Supporting Information

Figure S1 **Characterization of vole TS cell lines.** Morphology of the V1 TS cell line: undifferentiated state **(A)** and at 3–4 days of differentiation **(B)**. **(C)** RT-PCR analysis of TS cell markers in the V1 line. **(D)** Histological sections of the tumor formed by subcutaneous injection of vole TS cells into a *nude* mouse. Staining with hematoxylin-eosin. (1) necrosis zone, (2) proliferating zone, (3) giant cells.
(TIF)

Figure S2 **Distribution of repressive chromatin modifications in the V1 TS cells.** An example of repressive chromatin modification localization in the V1 TS cells. (**A**) uH2A (red); **(B)** HP1 (red); **(C)** H3K9me3 (red). Metaphase spreads were counterstained with DAPI (blue). The inactive X-chromosome (Xi) is indicated by arrow.
(TIF)

Table S1 **Results of a quantifications of fluorescence signals on Xi during vole TS differentiation.**
(DOC)
