# Supplementary figures and images for: Dynamics of the Two Heterochromatin Types during Imprinted X Chromosome Inactivation in Vole Microtus levis
Source: PLoS One. 2014 Feb 4;9(2):e88256. doi: 10.1371/journal.pone.0088256 (PMC3913780; doi:10.1371/journal.pone.0088256)

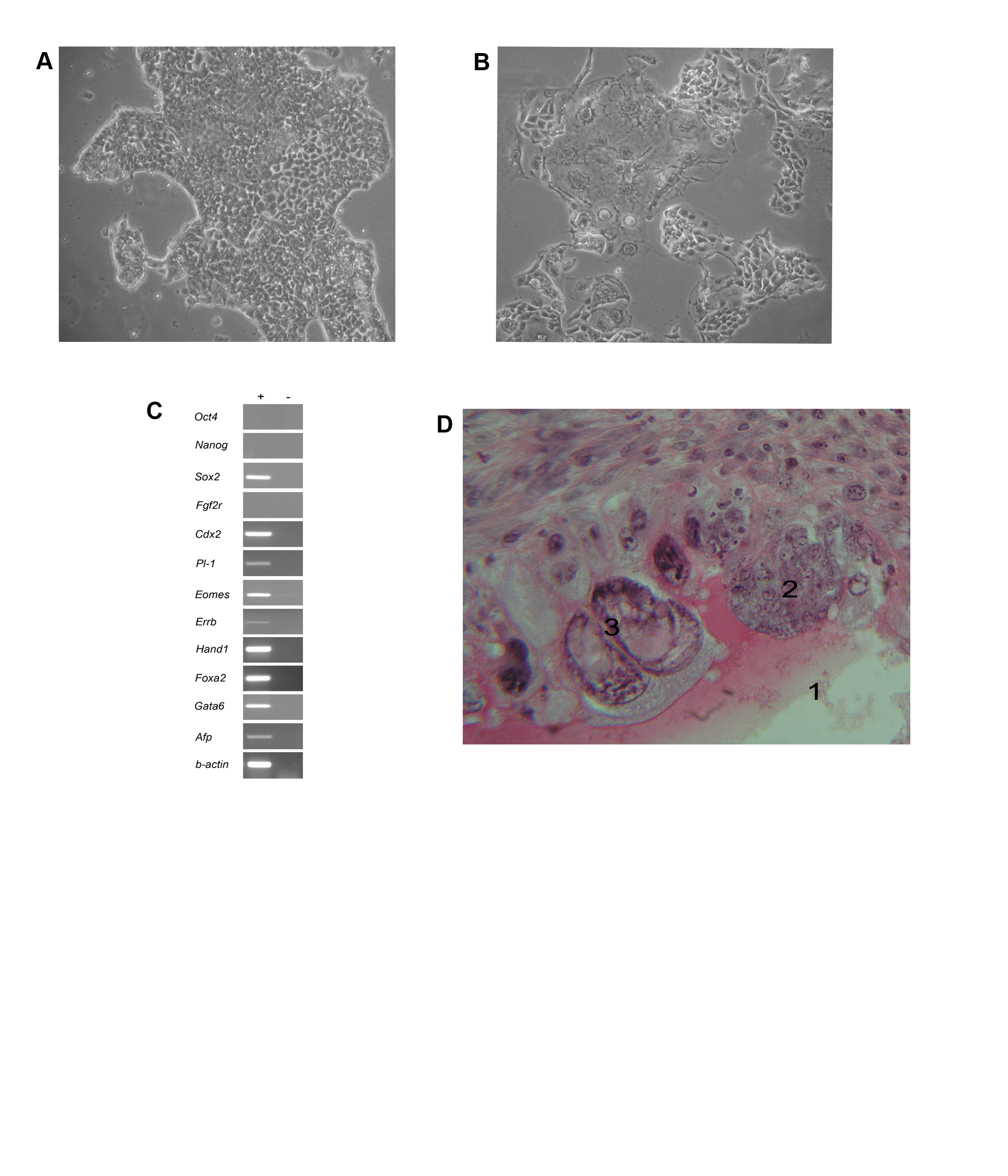

Supplement: Figure S1 — Characterization of vole TS cell lines. Morphology of the V1 TS cell line: undifferentiated state (A) and at 3–4 days of differentiation (B). (C) RT-PCR analysis of TS cell markers in the V1 line. (D) Histological sections of the tumor formed by subcutaneous injection of vole TS cells into a nude mouse. Staining with hematoxylin-eosin. (1) necrosis zone, (2) proliferating zone, (3) giant cells. (TIF) [file pone.0088256.s001.tif]

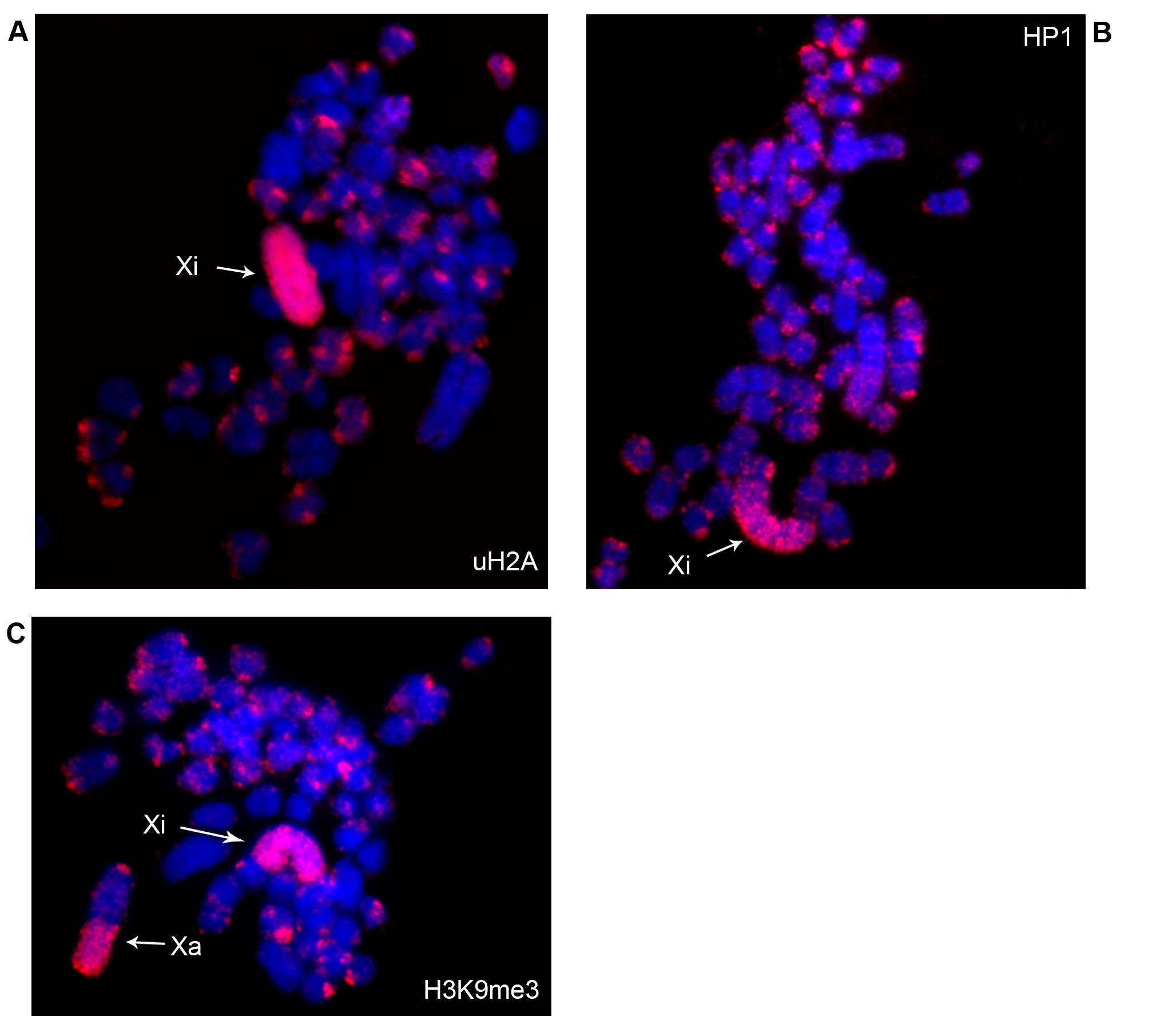

Supplement: Figure S2 — Distribution of repressive chromatin modifications in the V1 TS cells. An example of repressive chromatin modification localization in the V1 TS cells. (A) uH2A (red); (B) HP1 (red); (C) H3K9me3 (red). Metaphase spreads were counterstained with DAPI (blue). The inactive X-chromosome (Xi) is indicated by arrow. (TIF) [file pone.0088256.s002.tif]
