# Supplementary material for: Dynamics of the Two Heterochromatin Types during Imprinted X Chromosome Inactivation in Vole Microtus levis
Source: PLoS One. 2014 Feb 4;9(2):e88256. doi: 10.1371/journal.pone.0088256 (PMC3913780; doi:10.1371/journal.pone.0088256)
Supplement: Table S1 — Results of a quantifications of fluorescence signals on Xi during vole TS differentiation. (DOC) [file pone.0088256.s003.doc]

| Chromatin modifications | Day of differentiation  ( number of metaphases analyzed) | | | |
| --- | --- | --- | --- | --- |
|  | 0 | 2 | 4 | 6 |
|  | Total: 113 (100%) | Total: 98 (100%) | Total: 103(100%) | Total: 90 (100%) |
| Eed | 2 (1,77%) | 10 (10,2%) | 72 (69,9%) | 23 (25,5%) |
| H3K27me3 | 5 (4,42%) | 7 (7,14%) | 53 (51,4%) | 64 (71,1%) |
| H3K9me3 | 96 (85%) | 69 (70,4%) | 43 (41,7%) | 23 (25,5%) |
| uH2A | 87 (77%) | 72 (72,44%) | 66 (64,0%) | 70 (77,7%) |
| HP1 | 87 (77%) | 59 (60,2%) | 42 (40,7%) | 25 (27,7%) |
| H4K20me3 | 90 (79,6%) | 74 (75,5%) | 50 (48,5%) | 31 (34,4%) |
| No signals | 17 (15%) | 10 (10,2%) | 12 (11,6%) | 15 (16,6%) |
